# X-ray structure analysis of bacteriorhodopsin at 1.3 Å resolution

**DOI:** 10.1038/s41598-018-31370-0

**Published:** 2018-09-03

**Authors:** Nagayuki Hasegawa, Hideyuki Jonotsuka, Kunio Miki, Kazuki Takeda

**Affiliations:** Department of Chemistry, Graduate School of Science, Kyoto University, Sakyo-ku, Kyoto, 606-8502 Japan

## Abstract

Bacteriorhodopsin (bR) of *Halobacterium salinarum* is a membrane protein that acts as a light-driven proton pump. bR and its homologues have recently been utilized in optogenetics and other applications. Although the structures of those have been reported so far, the resolutions are not sufficient for elucidation of the intrinsic structural features critical to the color tuning and ion pumping properties. Here we report the accurate crystallographic analysis of bR in the ground state. The influence of X-rays was suppressed by collecting the data under a low irradiation dose at 15 K. Consequently, individual atoms could be separately observed in the electron density map at better than 1.3 Å resolution. Residues from Thr5 to Ala233 were continuously constructed in the model. The twist of the retinal polyene was determined to be different from those in the previous models. Two conformations were observed for the proton release region. We discuss the meaning of these fine structural features.

## Introduction

Bacteriorhodopsin (bR) is a light-driven proton pump found in the plasma membrane of a halophilic archaeon, *Halobacterium salinarum*^1,2^. bR is one of the most extensively studied membrane proteins, and has been investigated using a wide range of experimental and theoretical approaches. Its structure was first revealed by the electron diffraction method^3–5^. The bR molecule forms a trigonal 2D crystalline lattice called purple membrane (PM). Seven transmembrane helices (A–G) surround a retinal molecule which is covalently bound to the side chain of Lys216 in helix-G with the Schiff base (SB) linkage. The proton path is composed of charged residues such as Arg82, Asp85, Asp96, Glu194, Glu204, Asp212 and SB.

Following this initial structural characterization, numerous structures of bR and its homologues were determined by electron or X-ray diffraction methods at increasing resolution^6–9^. The highest resolution of 1.55 Å for the ground state bR (Protein Data Bank accession number 1C3W) was achieved using a tiny 3D crystal grown by the lipidic cubic phase (LCP) method^10^. However, radiation damage was not taken into consideration in that study, and some structures with low or no damage were reported thereafter^11–15^. These studies also elucidated that clear atomic movements occur under intense X-ray irradiation, especially around SB. In any case, individual atoms were not separately visualized on the electron density maps. Thus these approaches are not sufficient to elucidate the fine structural features critical to the color tuning and ion pumping properties. For this reason, the crystallographic structures are generally used for theoretical calculations only as the initial models.

bR is considered to have good potential as a nonlinear optical material^16–20^. In addition, bR homologues and their variants have begun to be utilized in optogenetics as a tool for artificial control of the neuronal activity by light^21,22^. In order to elucidate previously veiled features of the retinal chromophore as well as proton-path forming residues, we performed high-resolution X-ray crystallographic analysis of bR in the ground state. Our results will permit the rational design of new bR variants suitable for various applications.

## Results

### Assessment of X-ray damage

**Figure 1:**
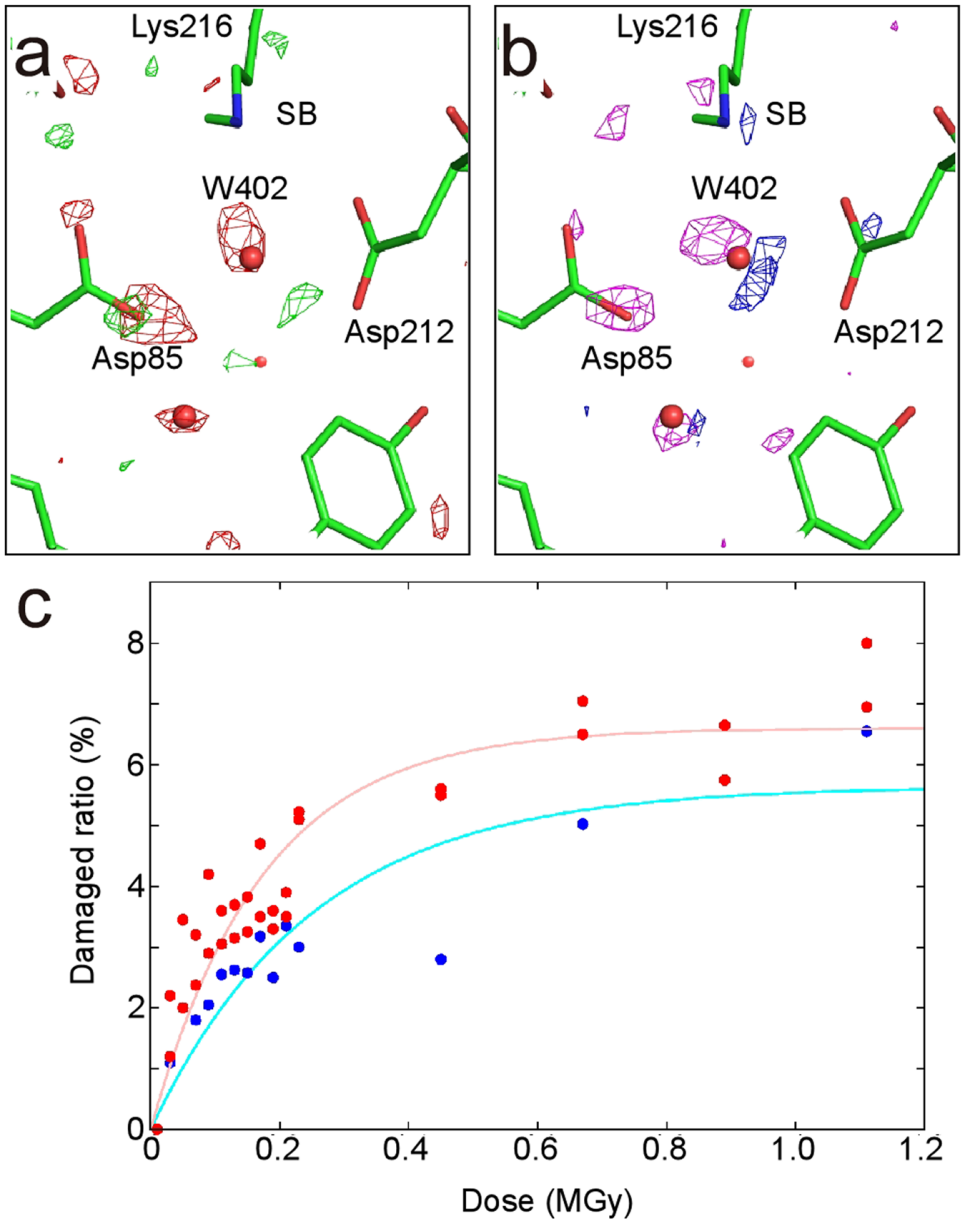
X-ray damage assessment of bR. (**a**) The difference Fourier map between the first (0.01 MGy) and damaged (0.45 MGy) datasets at 100 K. The positive (+4σ) and negative (−4σ) densities are represented as green and red meshes, respectively. (**b**) The difference Fourier map between the first (0.01 MGy) and damaged (0.45 MGy) datasets at 15 K. The positive (+4σ) and negative (−4σ) densities are represented as blue and pink meshes, respectively. (**c**) Changes of the damage ratio at 100 K (red) and 15 K (blue) as a function of the doses. Single exponential functions fitted to the occupancies are indicated as solid lines.

Large crystals with sizes of ~300 × 300 × 40 − 400 × 400 × 50 μm^3^ were obtained by the LCP method with high reproducibility by adding squalane and a detergent to the LCP host lipid (see the Methods section). The crystals were frozen after light adaptation for revealing the ground state structure with all-*trans* retinal. In order to compare the influence of X-rays at 15 K with that at 100 K, a series of diffraction datasets were successively collected from a single bR crystal at each temperature (Supplementary Tables 1 and 2). At 100 K, the difference Fourier maps showed many positive and negative peaks, especially around the proton path, as previously reported^13,14^ (Fig. 1a). At 15 K, difference peaks were also observed, and were localized at SB, Asp85 and Wat402 (Fig. 1b). According to the changes in the fraction of the damaged structure at the different doses, the damage was more suppressed at 15 K than at 100 K (Fig. 1c). The plot indicates that less than 5% of the damaged fractions were accumulated by the dose of 0.15 MGy at 15 K, while 5% were accumulated by the dose of 0.08 MGy at 100 K. Based on this assessment, we collected higher resolution datasets with the X-ray dose of ~0.1 MGy at 15 K in order to elucidate the intrinsic structural features of bR.

### High-resolution X-ray analysis

**Figure 2:**
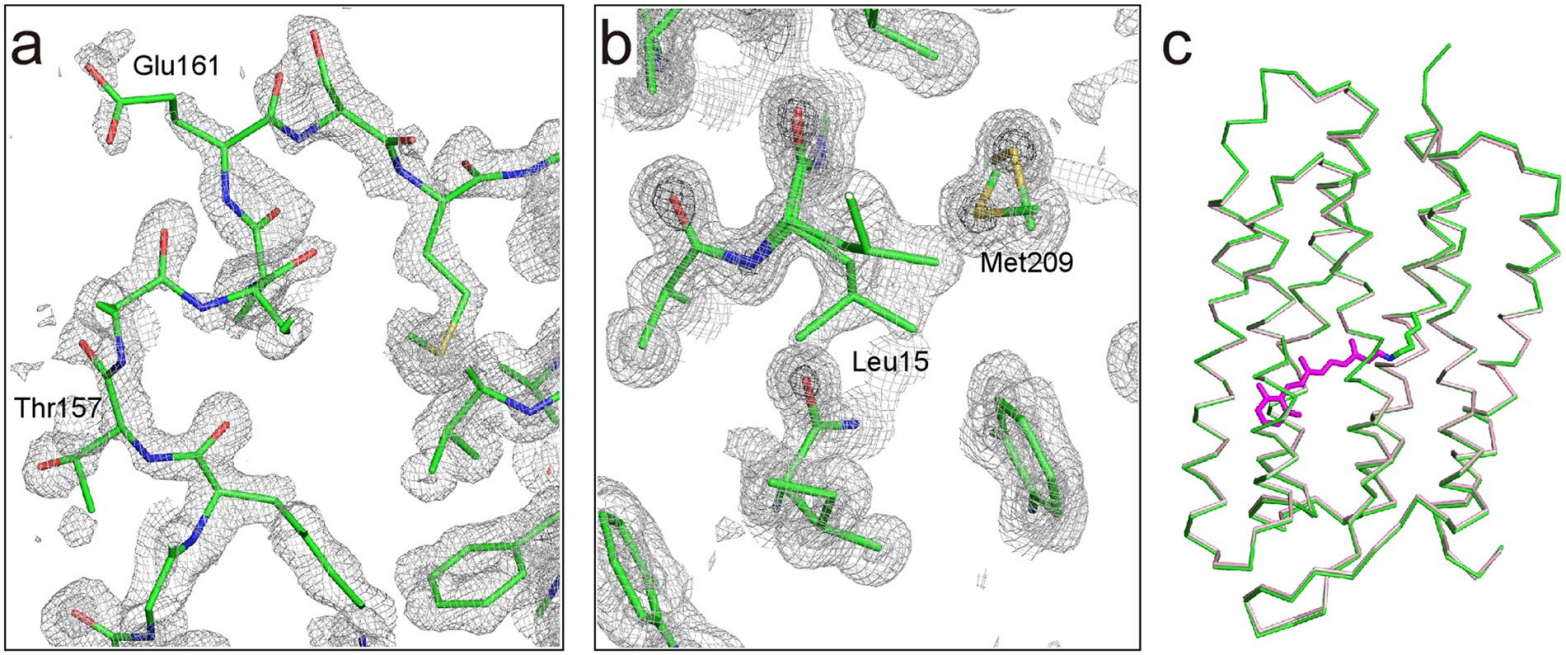
X-ray structure at 1.3 Å. (**a**) The 2*F*_obs_ − *F*_calc_ electron density map around the EF loop is shown at contour levels of 1.0σ (light gray) 3.0σ (gray) and 5.0σ (dark gray). (**b**) The map around the double conformational residues (Leu15 and Met209). (**c**) Superimposition with the previously reported structures. The structures of this study (green) and 1C3W (pink) are shown as Cα models. Retinal of this study is also shown in magenta.

A dataset with a resolution of 1.29 Å was obtained from a single crystal (Dataset I) (Table 1). The dose to each irradiated position in the dataset was estimated to be 0.1 MGy. In addition, two other datasets with 0.1 MGy better than 1.3 Å were composed using the data from plural isomorphous crystals (Dataset II and III). These high-resolution datasets enabled us to perform structural analyses using anisotropic displacement parameters with sufficient data to parameter ratio of ~3. As a result, we were able to obtain high quality electron density maps in which individual atoms were separately observed (Fig. 2a,b). The structures were almost identical to each other, with a root mean square deviation (rmsd) of ~0.1 Å in the superimposition of all Cα atoms. Therefore, we mainly present the structure from Dataset I in this paper, while geometric parameters are derived as the averaged values of the three structures. The structure consists of 229 residues, one retinal, four lipids and 30 waters (Table 1). The structure contains the loop EF (Thr157 − Glu161), which is absent in 1C3W^10^ (Fig. 2a). In addition, multiple conformations were observed for 8 residues (Fig. 2b). The rmsd value against 1C3W for common Cα atoms is 0.31 Å (Fig. 2c).

**Table 1:** Crystallographic and refinement statistics.

	Dataset I	Dataset II	Dataset III
**Data collection**			
No. of crystals	1	6	4
Dose (MGy/position)	0.10	0.10	0.10
Space group	*P*6_3_	*P*6_3_	*P*6_3_
Cell dimensions, *a*, *c* (Å)	60.59, 110.74	60.60, 110.71	60.63, 110.64
Resolution (Å)	50–1.29 (1.35–1.29)^a^	50–1.25 (1.28–1.25)	50−1.27 (1.30–1.27)
*R*_merge_^b^(%)	6.2 (94.4)	10.3 (308.7)	13.3 (433.1)
*I*/σ(*I*)	13.4 (1.8)	21.6 (1.7)	22.3 (1.1)
Completeness (%)	99.9 (99.7)	100.0 (100.0)	100.0 (100.0)
Redundancy	5.6 (5.5)	33.5 (33.4)	81.5 (81.5)
CC_1/2_ (%)	99.9 (48.6)	100.0 (50.2)	100.0 (51.0)
**Refinement**			
Resolution (Å)	50–1.29	50–1.25	50–1.27
No. of reflections	57755	63455	60573
Twin fraction	0.35	0.49	0.47
*R*_work_^c^/*R*_free_^d^ (%)	15.2/16.2	12.9/16.2	12.4/14.7
No. of atoms			
Protein	1834	1820	1806
Retinal	20	20	20
Lipid	166	166	120
Water	30.0	29.0	28.0
No. of multi-conformations	8	8	6

^a^Highest resolution shell is shown in parentheses.

^b^*R*_merge_ = Σ_hkl_Σ_i_|*I*_hkl,i_ − <*I*_hkl_>|/Σ_hkl_Σ_i_ *I*_hkl,i_.

^c^*R*_work_ = Σ_hkl_||*F*_obs_| − |*F*_calc_||/Σ_hkl_|*F*_obs_|.

^d^*R*_free_ was calculated by using 5% of the reflections that were not included in the refinement as a test set.

### Structure of retinal

**Figure 3:**
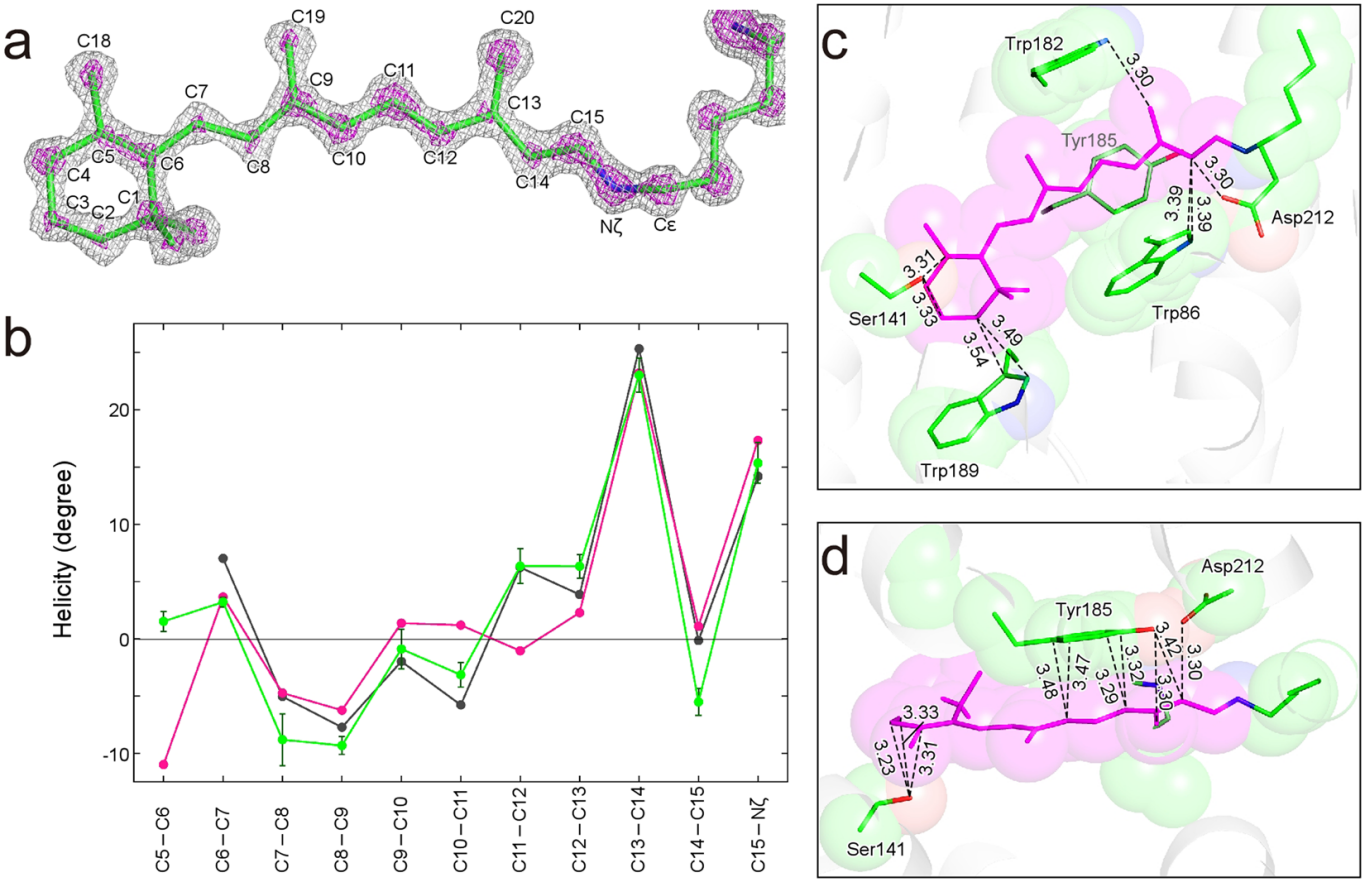
Detailed information for the retinal chromophore. (**a**) The *F*_obs_ − *F*_calc_ omit map of retinal and the side chain atoms of Lys216 is shown at contour levels of 4σ (gray) and 7σ (magenta). (**b**) Plot for the helicity angle along the polyene chain of retinal. The helicity for this study is plotted in green. Bars represent standard deviations derived from the three structures. In addition, helicities for 1C3W^10^ and a DFT/MM optimized structure^26^ are plotted magenta and gray, respectively. The helicity η, a twist from the planar conformation, is calculated with the relation, η(°) = (180 − torsion) × (−1)^n^ (ref.^26^). (**c**) Interactions between retinal and the protein environment. Side chains of residues within 3.5 Å from retinal are shown as semi-transparent spheres. (**d**) The top view of (**c**).

The delocalization of π electrons is directly involved in the properties of retinal, such as the absorption energy and p*K*_a_^23^. Therefore, there is strong demand for accurate crystallographic values for the torsion angles of the respective bonds for the polyene backbone of retinal, since the electronic structure is closely correlated with the planarity^23–27^. The high-resolution electron density map enables us to recognize the respective atomic positions of the retinal molecule which is in the all-*trans* conformation by means of light adaptation before freezing (Fig. 3a). The twists of the retinal polyene backbone are valued with a high accuracy of ~1° (Supplementary Table 3). C5–C6, C11–C12, and C14–C15 show large deviations of more than 5° from the structure in 1C3W (Fig. 3b). C5–C6 shows a small twist of 1.5°, while it shows a large twist of ~−10° in 1C3W. This twist may be due to the lack of resolution in the refinement calculations. On the other hand, a similar twist at C5–C6 was predicted for the isolated retinal molecule in the vacuum^23,25^. The nearly planar conformation at C5–C6 observed in this study may be achieved by interactions with surrounding residues at the β-ionone ring. The pattern of the twist along the polyene backbone in this study is more similar to that from the density functional theory with the molecular mechanics (DFT/MM) method than that in 1C3W^26^ (Fig. 3b). It is noteworthy that the twist at C11–C12 is already predicted in the DFT/MM optimized structure. This agreement verifies that the twist at the position is meaningful for retinal embedded in bR.

### Interactions between retinal and the surrounding residues

Some close contacts between retinal and the surrounding residues are observed in 1C3W, while interactions with the surrounding residues define the structural and electronic properties of the retinal molecule in bR. We measured these according to our more accurate structures. The retinal molecule interacts with Tyr185 by the π−π stacking configuration (Fig. 3c,d). A previous quantum chemical study pointed out that the electron transfer from Tyr185 to retinal is critical to the opsin shift^28^. The distance between C10 and Cδ2 of Tyr185 is longer by 0.10 Å than that in 1C3W. This difference is statistically significant because the value is four times larger than the standard deviation derived from the three structures. The difference is critical to the electron transfer reaction in proteins. Although the shortest interatomic distance of 3.13 Å was observed between C5 of retinal and Oγ of Ser141, the distance in this study was 3.31 ± 0.03 Å. In addition, the distances between Oγ of Ser141 and C3 and C4 of retinal were also longer than in 1C3W. The unusually close contacts in 1C3W may be caused by the distortion in the β-ionone ring structure.

### Hydrogen bonding around the SB linkage

**Figure 4:**
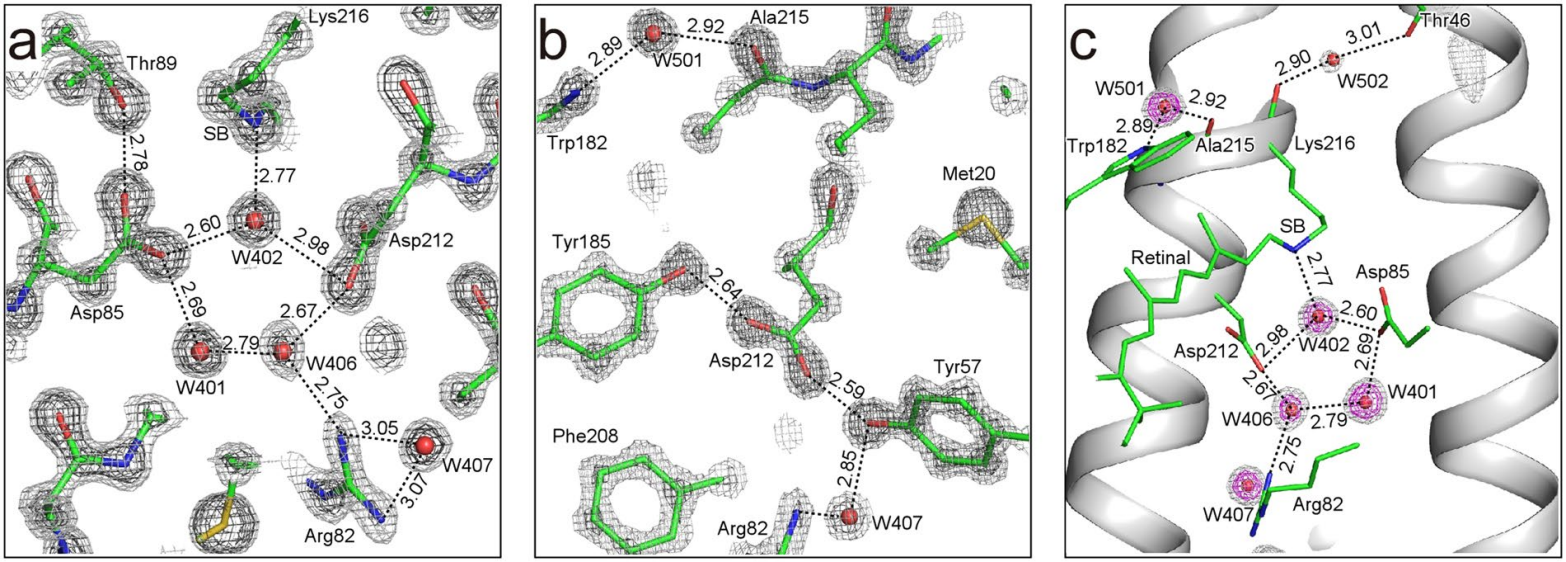
Hydrogen bonding pattern in the proton path. (**a**) Hydrogen bonding network between SB and Arg82. The 2*F*_obs_ − *F*_calc_ electron density map is shown at contour levels of 3σ (gray) and 5σ (dark gray). (**b**) Hydrogen bonding geometry around Tyr185 and Asp212. (**c**) The *F*_obs_ − *F*_calc_ omit map of water molecules in the proton path is shown at contour levels of 3σ (gray) and 5σ (magenta).

Hydrogen bonding in the proximity of the SB linkage realizes a proton switch that converts the isomerization of retinal to the vectorial movement of protons^29^. Therefore, there is great demand for accurate geometries around the linkage. In this study, distances without any restraints were successfully obtained from the high-resolution X-ray analyses (Fig. 4 and Supplementary Table 4). It is known that the distance between the Nζ atom of SB and Water 402 is elongated as a result of X-ray damage^14^. The average distance in our three structures was 2.77 ± 0.03 Å. This confirms that X-ray damage was suppressed to a negligible level. On the other hand, the distance in 1C3W is 2.87 Å and substantially larger by ~ 0.10 Å than ours, indicating the structure is influenced by X-rays. Differences between 1C3W and our present structure greater than 0.1 Å were also observed for the hydrogen bond distances between Asp85 Oδ2 and W406, Asp85 Oδ2 and W401, Thr89 Oγ1 and Asp85 Oδ1, Asp212 Oδ2 and W402 (Supplementary Table 4). This implies that the carboxyl groups involved in these hydrogen bonds are decarboxylated in 1C3W beyond a negligible level.

### Multi-conformations in the proton release region

**Figure 5:**
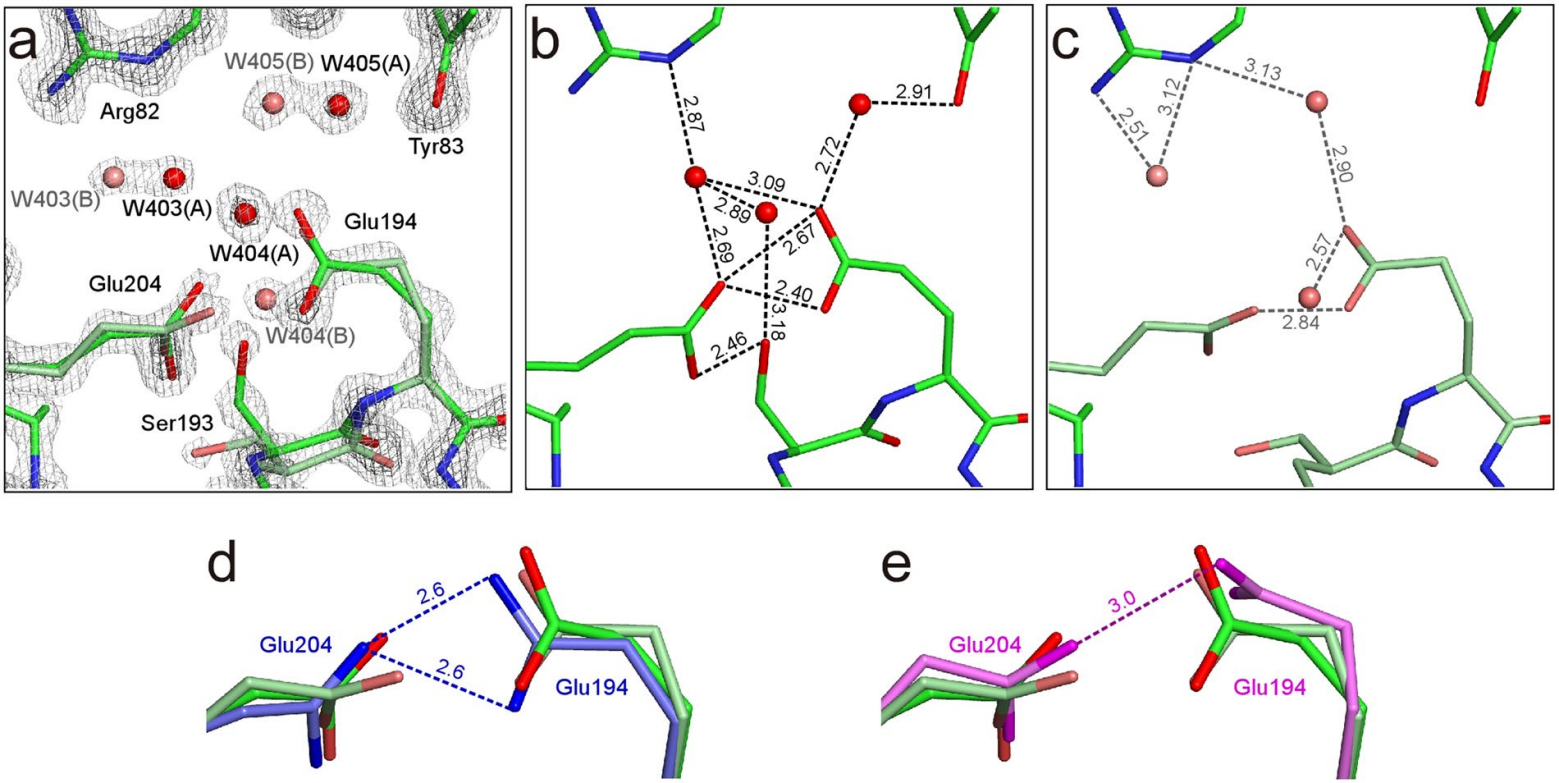
Multi-conformations around the proton release group. (**a**) The 2*F*_obs_ − *F*_calc_ map is shown at contour levels of 2σ (gray) and 4σ (dark gray). (**b**) Hydrogen bonds shorter than 3.2 Å for the major conformation are represented as black dotted lines. (**c**) Hydrogen bonds for the minor conformation are represented as gray dotted lines. (**d**) Comparison of the conformations of Glu194 and Glu204 with those in 1IW6 (blue). (**e**) Comparison with 1C3W (pink).

The proton release region of bR, which consists of two glutamate residues, shows unique properties such as high p*K*_a_ of ~9.7 and the continuum IR band even in the ground state^30–34^. Our study reveals that the side chain of Glu204 in the proton release group at the extracellular side of bR takes two conformations (Fig. 5a–c). The occupancies of the two conformations are refined to be 0.55 and 0.45. The distances between Glu194 and Glu204 are 2.40 ± 0.04 and 2.67 ± 0.06 Å for the major conformation. The major conformation is almost identical to that in 1IW6 (Fig. 5d), while the minor one resembles that in 1C3W (Fig. 5e). The value of 2.40 ± 0.04 Å is significantly shorter than the standard distance of hydrogen bonds of ~2.8 Å. Such a short distance at the site has been reported for an analogous protein, archaerhodopsin-2, where the distance is 2.3 Å^35^. Water molecules, Wat403, Wat404 and Wat405, also have two double positions in relation to the double conformation of Glu194 and Glu204 (Fig. 5b,c). It has been suggested that the delocalizations of protons in the region are critical to proton storage. The delocalization of protons can generate multiple structures to the region^34^. The double conformations in our structure may correspond to these.

## Discussion

Hydrogen bonding is critical to the stability and function of proteins. There is thus great demand for the accurate geometries of hydrogen bonds, especially around the SB linkage. In this study, we determined an unprecedentedly high-resolution structure of bR from a low-damage electron density map in which the individual atoms were separately visualized. Consequently, distances between the hydrogen bonding donor and acceptor atoms were refined without any restraints, and their errors were estimated by comparing the three structures refined at a resolution better than 1.3 Å. The three structures exhibited some notable differences from the structure of 1C3W in terms of the hydrogen bonding distances. Some of the hydrogen bonds around the SB have interesting features, as discussed below.

As reported previously, water molecules in the proton path do not show the tetrahedral coordination that usually occurs in protein surfaces as well as bulk waters. Several models have been proposed for the manner of hydrogen bonding of the water molecules near SB^36–38^. However, none of them have been fully accepted. Our precise structure allows a more plausible picture of the interactions around SB, although it still does not include any of the hydrogen atoms. One of the hydrogen atoms of Wat402 doubtlessly interacts with Oδ2 of Asp85 based on the geometrical aspects. However, another hydrogen atom of Wat402 seems not to form a hydrogen bond with Oδ2 of Asp212, because the lone pair electrons of Oδ2 may be geometrically inappropriate as a hydrogen bonding partner. Instead, π electrons of the carboxyl group of Asp212 may interact with the second hydrogen atom of Wat402. In this case, the interaction can be enhanced due to the partial positive charge of the hydrogen induced by the electronic polarization of O−H of Wat402. On the other hand, one of the lone pairs of Wat402 could plausibly interact with Nζ of SB. The fourth interaction of Wat402 in not found in the crystal structure, while the tetrahedral coordination is expected to be stable. Such incomplete coordination is found for other waters in the proton path of bR (Fig. 4c), and may be important for the proton transfer reaction. The free bonds could more easily be involved in the proton release and acceptance than those in completely coordinated waters. The tetrahedral coordination of water is very stable and strongly related to its unique properties^39^. In contrast, the non-tetrahedral coordination of the waters in the proton path of bR may store energy and play a key role in proton pumping.

Two protonated aspartate residues, Asp96 and Asp115, exist in bR in the ground state, in which the side chains interact with Thr46 and Thr90, respectively. The hydrogen bonding distances are 2.66 ± 0.01 and 2.50 ± 0.03 Å, respectively. The distances are significantly (~0.1 Å) longer than those in 1C3W. The distances in this study are adequate as normal hydrogen bonds, while it has been indicated that the short distances give the strongest interactions among the inter-helical interactions in bR^40^. On the other hand, an MD simulation analysis indicates that the two interactions are unexpectedly dynamic and longer than those in 1C3W^41^. Accordingly, inter-helical interactions, which define functional motions as well as stability, should be reevaluated based on our structure.

In addition to the classical hydrogen bonds, CH···O type hydrogen bonds are frequently found in protein structures^42–44^. A CH···O type hydrogen bond may be formed between the C14 of retinal and Oδ1 of Asp212 with a distance of 3.30 Å (Fig. 3c). This interaction influences the color tuning through the charge distribution on the polyene of retinal. The resulting C14–H···Oδ1 angle is ~130°, if the standard C–H bond length of 1.0 Å is assumed for the putative hydrogen atom on C14. The dissociation energy (*D*_e_) for similar cases is 5 ~ 10 kJ/mol in quantum chemical and topological analyses of high resolution crystal structures^43,45,46^. The value is intermediate between the classical hydrogen bond and van der Waals interaction.

In conclusion, our bR structure at an unprecedented resolution of higher than 1.3 Å provides detailed structural information, especially for retinal and the proton path. Because differences on the order of 0.1 Å are critical to proton transfer and charge transfer in proteins, the small differences between this study and the previous structures are significant to investigations of bR. The X-ray crystallographic information will make a critical contribution to elucidation of the ion pumping mechanism of retinal proteins in combination with computational and spectroscopic approaches.

## Methods

### Preparation of crystals

PM was purified from *Halobacterium salinarum* strain R1 (JMC9409) by a standard procedure^1^. Crystals were obtained by the LCP method^47^ with some modification. Briefly, squalane (Sigma-Aldrich) and trehalose C16 (Dojin) were added to the monoolein-based LCP at final concentrations of 1.0% and 5.0%, respectively. 20 μL of bR solution and 32 μL of the LCP matrix were mixed with two syringes (Hamilton) coupled with a connector^48^. Each LCP drop (2 μL) was immersed in 30 μL of 1.8 M Na/K phosphate (pH 5.6) in a microbridge (Hampton Research). The microbridge was located with 500 μL of 2.0−2.5 M Na/K phosphate (pH 5.6) in a 24-well crystallization plate. Crystals were grown to a typical size of 300 × 300 × 40 − 400 × 400 × 50 μm^3^ after more than 3 months (Supplementary Fig. 1a). The LCP containing matured crystals was equilibrated to 2.5 M Na/K phosphate for ~24 h. The LCP matrix around crystals was removed by washing with squalane oil. The squalane oil was also used as a cryoprotectant. The crystals were picked up with nylon loops and flash cooled with a nitrogen gas stream at 100 K after illumination with white halogen light for ~1 min. The frozen crystals were transferred to liquid nitrogen while avoiding light, and stored in a liquid nitrogen tank.

### X-ray data collection

All diffraction experiments were performed at BL41XU of SPring-8 (Hyogo, Japan). Crystals were cooled at 15 K or 100 K with a helium gas stream on the diffractometer. Crystal centering was performed under ambient room light, while the light was turned off during data collection (Supplementary Fig. 1b,c). The wavelength of X-rays and the beam size were set to 0.80 Å and 30 × 20 μm^2^, respectively. Aluminum attenuators were used to reduce X-rays of the initial flux (8 × 10^12^ photons·sec^−1^) because the flux was too high. The crystal-to-detector distance was set to 210 mm. Diffraction spots were recorded with a Pilatus 6 M detector (Dectris). The helical data-collection method was employed in order to suppress the dose for each irradiated position.

For the assessment of X-ray damages, twelve successive datasets (from 0.01 to 0.23 MGy) were successively collected with attenuated X-rays. High dose irradiations (0.22 MGy each) were performed before data collections of the 13th to 16th datasets in order to inflict damage. The doses were estimated with the program RADDOSE^49^, and are listed in Supplementary Tables 1 and 2.

### Data analysis

Diffraction images were integrated and processed with the program XDS^50^. The resolution limits were defined at CC_1/2_ ~0.5 (ref.^51^). The structures were solved by the molecular replacement method using 1C3W as an initial model^10^. The initial simulated annealing refinement from 2500 K with isotropic *B* factors was carried out using the program CNS^52^. The structures were monitored and corrected with the program COOT^53^ according to the 2*F*_obs_ − *F*_calc_ and *F*_obs_ − *F*_calc_ maps. Further refinements with the anisotropic *B* factors were performed with the program SHELX^54^. The refinement statistics are listed in Table 1. The structures were validated with the program Molprobity^55^. The molecular structures presented in the figures were made with the program PyMOL^56^. The change in the damage ratio was estimated from the occupancies in the refinement using the method described previously^14^. The damaged structure was from 1MD1^14^. However, the damage ratio (*q*) could be refined only for datasets with doses higher than 0.15 MGy. Therefore, we additionally refined all structures by using the single structure and calculated the average of atomic *B* factor 〈*ΔB*〉 of four atoms, Oδ1 and Oδ2 of Asp85, Nζ of Lys216 and Wat402 showing the largest increase in the atomic *B* factors. The higher dose datasets, which were refined by the two procedures, allowed us to derive the relation, *q* = 6.7 × 〈*ΔB*〉. Accordingly, we used the relation for the estimation of the damage ratio in the lower dose datasets. The damage ratio was fit to a single exponential function for each temperature.

### Data Availability

The coordinates and structure factors have been deposited in the Protein Data Bank under accession numbers 5ZIL (for Dataset I), 5ZIM (for Dataset II) and 5ZIN (for Dataset III).

## Electronic supplementary material

Supplementary information
