# Supplementary material for: X-ray structure analysis of bacteriorhodopsin at 1.3 Å resolution
Source: Sci Rep. 2018 Sep 3;8:13123. doi: 10.1038/s41598-018-31370-0 (PMC6120890; doi:10.1038/s41598-018-31370-0)
Supplement: Supplementary file 1 — Supplementary information [file 41598_2018_31370_MOESM1_ESM.pdf]

Supplementary Information

X-ray structure analysis of bacteriorhodopsin at 1.3 Å resolution

Nagayuki Hasegawa, Hideyuki Jonotsuka, Kunio Miki and Kazuki Takeda*

Department of Chemistry, Graduate School of Science, Kyoto University, Sakyo-ku, Kyoto 606-8502, Japan

*Correspondence email: ktakeda@kuchem.kyoto-u.ac.jp

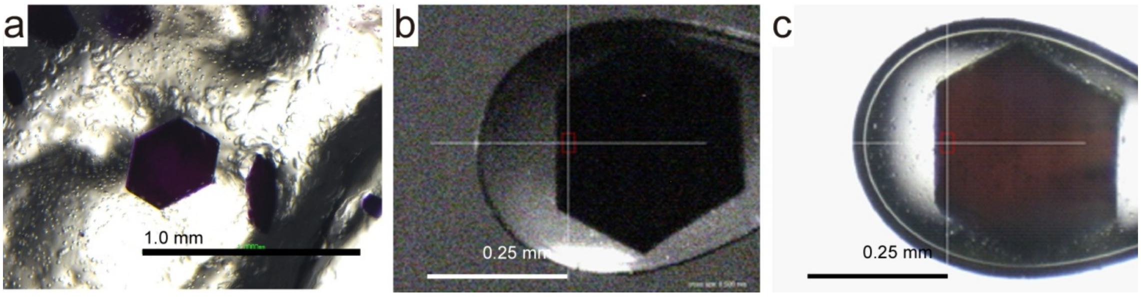

Supplementary Figure 1. Crystal of bR.

(a) A large crystal in the LCP matrix. The scale bar indicates 1.0 mm. (b) A crystal before X-ray irradiation. The photograph was taken under ambient room light. The scale bar indicates 0.25 mm. (c) The same crystal after X-ray irradiation with a dose of 1.1 MGy at 100 K. The photograph was taken using a white LED light.

Supplementary Table 1. Data collection and crystallographic statistics for datasets for the radiation damage assessment at 100 K.

Data#	Dose (MGy)	Cell constant a, c (Å)	$I/\sigma(I)$	R_{sym} [a] (%)	$CC_{1/2}$ (%)	Wilson B ($Å^2$)
1-01	0.01	60.64, 110.45	1.6	89.1	49.8	16.5
1-02	0.03	60.65, 110.46	1.6	88.1	50.1	16.3
1-03	0.05	60.65, 110.46	1.6	88.4	48.2	16.2
1-04	0.07	60.65, 110.46	1.6	89.2	48.6	16.2
1-05	0.09	60.65, 110.47	1.6	88.5	48.5	16.0
1-06	0.11	60.65, 110.47	1.6	88.5	50.0	16.1
1-07	0.13	60.64, 110.47	1.6	88.8	49.2	16.1
1-08	0.15	60.64, 110.47	1.6	88.6	47.7	16.1
1-09	0.17	60.64, 110.47	1.6	88.4	47.5	16.1
1-10	0.19	60.64, 110.47	1.6	89.9	49.9	16.1
1-11	0.21	60.64, 110.47	1.6	89.3	46.8	16.1
1-12	0.23	60.64, 110.47	1.6	89.4	49.0	16.1
1-13	0.45	60.64, 110.48	1.6	91.2	47.3	16.3
1-14	0.67	60.64, 110.48	1.6	90.4	48.0	16.3
1-15	0.89	60.64, 110.49	1.5	93.5	46.0	16.3
1-16	1.11	60.64, 110.49	1.5	94.1	46.9	16.3
2-01	0.01	60.61, 110.68	1.6	84.4	50.9	15.2
2-02	0.03	60.62, 110.69	1.6	84.8	50.9	15.0
2-03	0.05	60.62, 110.69	1.6	84.2	51.0	14.9
2-04	0.07	60.62, 110.69	1.6	85.0	50.6	15.0
2-05	0.09	60.65, 110.69	1.6	85.4	50.7	14.9
2-06	0.11	60.62, 110.69	1.6	85.0	51.1	15.0
2-07	0.13	60.62, 110.69	1.6	85.1	50.5	15.0
2-08	0.15	60.62, 110.69	1.6	85.0	51.1	15.0
2-09	0.17	60.62, 110.69	1.6	84.4	51.7	15.0
2-10	0.19	60.62, 110.69	1.6	85.8	49.5	15.0
2-11	0.21	60.62, 110.69	1.6	85.6	51.6	15.0
2-12	0.23	60.62, 110.69	1.6	84.7	50.7	15.0
2-13	0.45	60.61, 110.70	1.6	85.6	50.2	15.1
2-14	0.67	60.61, 110.70	1.6	87.4	48.2	15.2
2-15	0.89	60.61, 110.71	1.6	88.9	47.0	15.2
2-16	1.11	60.61, 110.71	1.5	89.1	49.7	15.3

The highest resolution shells for 1-01 to 1-16 are 1.69–1.57 Å, while those for 2-01 to 2-16 are 1.69–1.55 Å. The $I/\sigma(I)$, R_{sym} and $CC_{1/2}$ values are for the highest resolution shells.

[a] $R_{sym} = \Sigma_{hkl}\Sigma_i | I_{hkl,i} - \langle I_{hkl} \rangle | / \Sigma_{hkl}\Sigma_i I_{hkl,i}$.

Supplementary Table 2. Data collection and crystallographic statistics for datasets for the radiation damage assessment at 15 K.

Data#	Dose (MGy)	Cell constant *a*, *c* (Å)	$I/\sigma(I)$	R_{sym}[a] (%)	$CC_{1/2}$ (%)	Wilson *B* (Å^2)
3-01	0.01	60.59, 110.53	1.8	77.6	50.7	15.6
3-02	0.03	60.59, 110.53	1.8	77.8	53.5	15.5
3-03	0.05	60.59, 110.53	1.8	77.1	54.2	15.5
3-04	0.07	60.59, 110.53	1.8	77.7	53.8	15.4
3-05	0.09	60.59, 110.53	1.8	78.7	51.4	15.6
3-06	0.11	60.59, 110.53	1.8	78.2	52.5	15.4
3-07	0.13	60.59, 110.53	1.8	79.0	53.0	15.6
3-08	0.15	60.59, 110.53	1.7	78.5	52.6	15.6
3-09	0.17	60.59, 110.53	1.8	77.9	53.5	15.6
3-10	0.19	60.59, 110.53	1.8	78.2	51.1	15.7
3-11	0.21	60.59, 110.53	1.8	77.6	53.1	15.5
3-12	0.23	60.59, 110.53	1.8	78.0	51.8	15.6
3-13	0.45	60.59, 110.54	1.8	77.9	51.5	15.6
3-14	0.67	60.59, 110.54	1.7	78.6	51.3	15.7
3-15	0.89	60.59, 110.54	1.7	80.8	51.1	15.7
3-16	1.11	60.59, 110.55	1.7	81.4	50.2	15.8

The highest resolution shells for 3-01 to 3-16 are 1.69–1.59 Å. The $I/\sigma(I)$, R_{sym} and $CC_{1/2}$ values are for the highest resolution shells.

[a] $R_{sym} = \Sigma_{hkl}\Sigma_i | I_{hkl,i} - \langle I_{hkl}\rangle | / \Sigma_{hkl}\Sigma_i I_{hkl,i}$.

Supplementary Table 3. Dihedral angle for the polyene chain of retinal.

Bonds	Dataset I	Dataset II	Dataset III	Average	s.d.*
C5–C6	1.2	0.9	2.5	1.5	0.9
C6–C7	2.8	3.2	3.6	3.2	0.4
C7–C8	-8.4	-6.8	-11.3	-8.8	2.3
C8–C9	-10.2	-8.9	-8.8	-9.3	0.8
C9–C10	0.9	-2.5	-1.1	-0.9	1.7
C10–C11	-4.3	-2.1	-3.0	-3.1	1.1
C11–C12	4.7	6.9	7.5	6.4	1.5
C12–C13	6.5	7.3	5.2	6.3	1.0
C13–C14	23.9	23.8	21.3	23.0	1.5
C14–C15	-6.9	-4.7	-5.0	-5.5	1.2
C15–Nζ	15.0	13.8	17.3	15.4	1.8

*Standard deviation for the three datasets.

Supplementary Table 4. Hydrogen bonding distances around SB.

Hydrogen bonding pair			Dataset I	Dataset II	Dataset III	Average	s.d.*
Lys216 Nζ	–	W402	2.78(4)	2.79(3)	2.73(2)	2.77	0.03
Wat402	–	Asp85 Oδ2	2.60(3)	2.58(2)	2.62(2)	2.60	0.02
Wat401	–	Asp85 Oδ2	2.69(3)	2.70(2)	2.67(2)	2.69	0.02
Wat401	–	W406	2.78(4)	2.76(3)	2.82(2)	2.79	0.03
Wat406	–	Asp212 Oδ2	2.63(4)	2.70(3)	2.67(2)	2.67	0.04
Wat402	–	Asp212 Oδ2	2.99(3)	2.98(2)	2.98(2)	2.98	0.01
Thr89 Oγ1	–	Asp85 Oδ1	2.78(3)	2.78(2)	2.77(2)	2.78	0.01
Arg82 Nη	–	W406	2.75(7)	2.83(6)	2.69(5)	2.75	0.06

Values in parentheses are the estimated standard deviations derived from the full-matrix least squares refinement.

*Standard deviation for the three datasets.
